# The Year-Book of Treatment for 1892

**Published:** 1892-05

**Authors:** 


THE YEAR-BOOK OF TREATMENT FOR 1892. A Critical Review for Practitioners of Medicine and Surgery. Philadelphia: Lea Brothers & Company. 1892.

Again are we called upon to notice this valuable little annual, and we cannot do the profession better service than to say that year by year it appears with improved and still more valuable material. The practitioner who leads an active life, especially in the country, cannot always put his hand upon every journal or volume that issues from the press, and, consequently, some condensed book, grouping together the salient features of treatment that have been brought out during the year, becomes a necessity. The one before us easily supplies the want named, and we recommend it to all who need to consult such a work.

